# Two-stage Omental Flap Approach for Ascending Aortic Graft Infection

**DOI:** 10.21470/1678-9741-2020-0040

**Published:** 2020

**Authors:** Darío Andrade, Eric E. Vinck, Laura Niño Torres

**Affiliations:** 1 Department of Cardiovascular Surgery, Fundación Clínica Shaio, Bogota, Colombia.; 2 Department of Surgery, Universidad del Rosario, Bogota, Colombia.

Ascending aortic graft (AAG) infection is considered a disastrous complication and a serious challenge for cardiovascular surgeons. When accompanied by mediastinitis, it poses a serious threat to the patient^[1,2]^. AAG has an incidence of 1-2%, and the mortality is high as 25-88%^[3,4]^. For a long time, it has been considered as one of the few inoperable complications of cardiovascular surgery^[1]^. Currently, there is little evidence on the optimal approach to this life-threatening complication, however, omental flaps may be considered as a promising option. Here we present an example.

A 68-year-old female patient, with clinical history of a supracoronary tube graft prosthesis due to a type A aortic dissection a year before, presented with wound dehiscence and foreign body reaction. She was taken to surgery for wire removal. Ten months later she consulted presenting a non-fetid wound secretion associated with sternal hemorrhage. An aorto-cutaneous fistula was suspected and a chest computed tomography showed a periaortic hematoma with anastomotic leak, mediastinal widening, and an aortic maximum diameter of 36 mm. A 7-cm pseudoaneurysm of the proximal anastomosis in close relation with the sternum in communication with the skin was found. A second 6-cm pseudoaneurysm in the distal anastomosis with signs of infection was also found. The infected aortic graft was removed with extensive debridement, and aortic prosthesis replacement was performed (Maquet Intergard Silver) (Figure 1A and B). Circulatory arrest time was 40 min, total cardiopulmonary bypass time was 200 min, including 45 minutes of ischemia. Mediastinal packing was performed, and the patient was sent to the intensive care unit (ICU). For the second step of the procedure 72 hours later (delayed approach), mediastinal lavage and omental flap translocation were done (Figure 1C and D). Omental flap pedicle isolation was performed by freeing an 8-10 cm wide strip off the transverse colon, keeping it attached to the stomach by the right gastroepiploic artery with perfusion through the arc of Barkow and passed through a retrosternal tunnel to the front of the pericardium (Figure 2A and B). Blood cultures were positive for *Candida parapsilosis*. The patient had a successful recovery and was eventually discharged. At one-year follow-up, the patient has not required reintervention and is in good health.

**Fig. 1:**
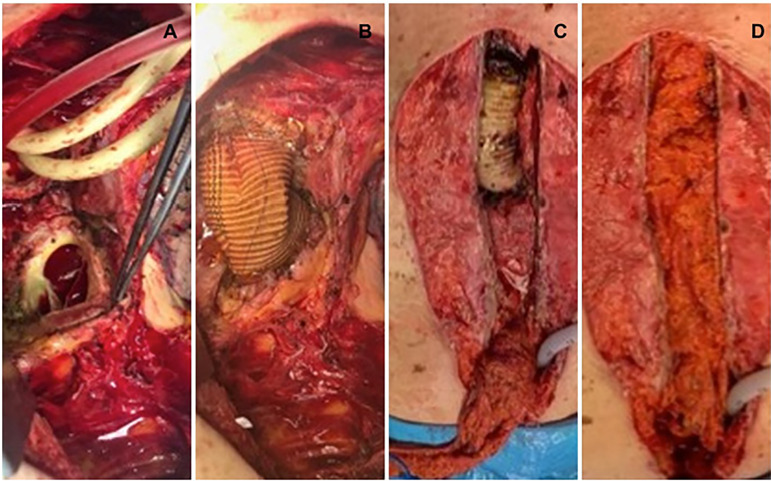
Stage 1: A) Excision of the infected graft, cerebral perfusion (Foley catheters). Aortic root (forceps). B) Re-grafting. Stage 2: C) Omental flap translocation though the diaphragm to the anterior mediastinum. D) Omental wrapping of the graft.

**Fig. 2:**
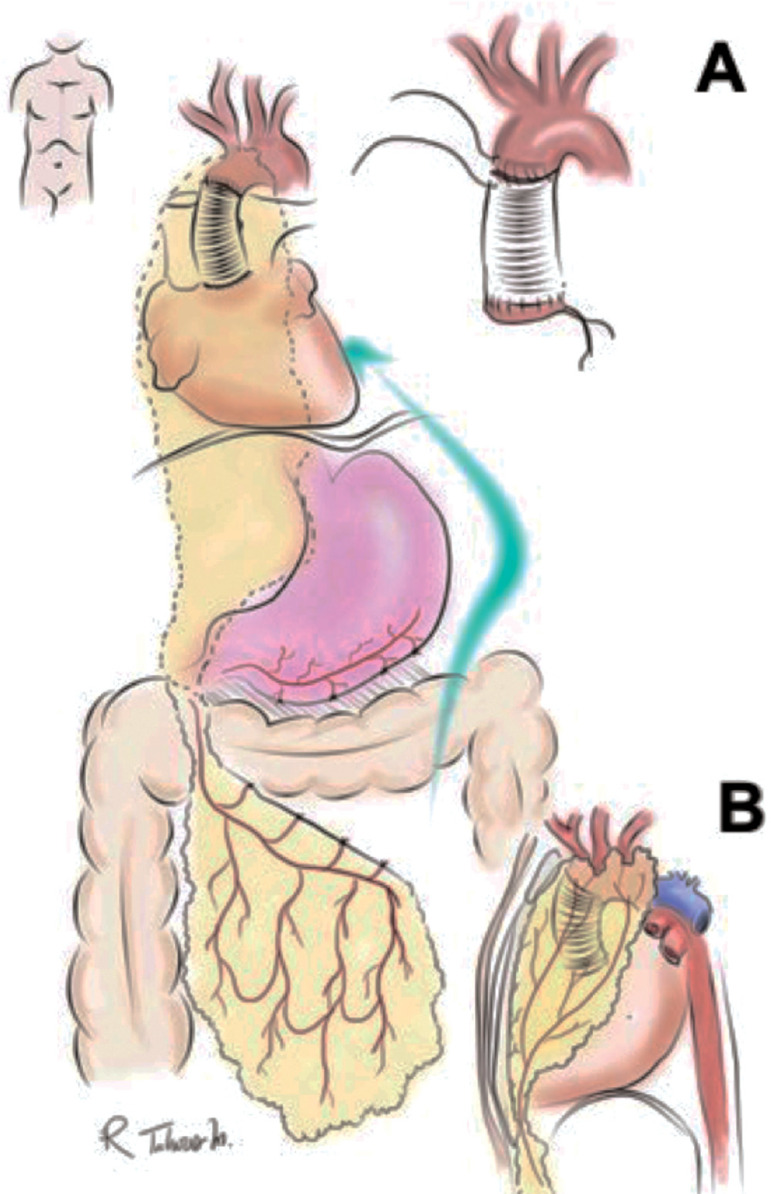
Schematic for omental flap translocation. A) Coronal view of omental flap translocation. B) Sagittal view of omental flap translocation to the anterior mediastinum.

## FINAL COMMENTS

Aortic graft infections can be classified into early, within three months, and late presentations, being associated to the causative pathogen^[4]^. Early infection is related to *Staphylococcus aureus* and Gram-negative bacterial infection. The ideal treatment for ascending aortic prosthesis infection remains controversial and options are limited because of both anatomic and physiologic considerations^[1-3]^. Hargrove and Edmunds proposed definitive management principles: wide debridement of infected tissues including vessel wall, viable tissue transposition (omentum or muscle), drains, long-term antibiotic administration, and graft excision^[3,4]^. A combination of these is a two-step approach with aortic prosthesis replacement, extensive debridement, and antiseptic lavage in the first stage, followed by an omental flap wrapping of the new graft in a second step 24-72 hours later^[5-7]^. Antibiotic therapy only is rarely effective, with 43% of patients requiring surgery, justifying aggressive initial surgical treatment. Tissue flaps should be used to obliterate dead space, provide vascularized tissue, and provide vascularized coverage of the graft. The omentum also has antibacterial effects with immunologically active cells, cellular proliferation, fibrous tissue formation, and adhesion. It also absorbs wound secretion, eliminating substrates for bacterial growth. Extensive vascularization and neovascularization potentially increase the blood supply, leading to higher antibiotic concentration at the infection site^[5-7]^. Potential for graft infection remains long after the original operation - up to 40% after two or more years. Surgery for omental flap transposition requires a stable patient, therefore a two-step approach should be considered for critical patients^[4-7]^. Complications of omental flaps include impaired blood supply resulting in necrosis, spread of intrathoracic infection to the abdomen, and diaphragmatic herniation. Close surveillance for infection recurrence or formation of a false aneurysm remains mandatory.

AAG infection is a life-threatening complication. There are many different surgical options available and they should be tailored to the individual patient; in general, graft resection should always be attempted. For high-risk and critical patients, a twostage thoracic omental flap transposition is a feasible procedure for treating serious infections after aortic graft surgery due to its anatomic and physiologic properties. This two-step approach looks promising due to the elimination of the infected graft and allows for the omental flap transposition following ICU patient stabilization.

## References

[1] Krabatsh T, Hetzer R (1995). Infected ascending aortic prothesis: successful treatment by thoracic transposition of the greater omentum. Eur J Cardio-thoracic Surg.

[2] Luciani N, Lapenna E, De Bonis M, Possati GF (2001). Mediastinitis following graft replacement of the ascending aorta: conservative approach by omental transposition. Eur J cardio-thoracic Surg.

[3] Hargrove WC, Edmunds LH (1984). Management of Infected Thoracic Aortic Prosthetic Grafts. Ann Thorac Surg.

[4] Bianco V, Kilic A, Gleason TG, Arnaoutakis GJ, Sultan I (2018). Management of thoracic aortic graft infections. J Card Surg.

[5] Coselli J, Cuyeyt K, LeMaire S (1999). Management of thoracic aortic graft infections. Ann Thorac Surg.

[6] Aspern K von, Etz CD, Mohr FW, Battellini RR (2016). Two-Stage Procedure for Infected Aortic Graft Pseudoaneurysm: 10-Year Follow Up after Omental Prosthesis Wrapping. Aorta.

[7] Hernandez JA, Stranix JT, Piwnica-Worms W, Azoury SC, Kozak GM, Grimm JC, Vallabhajosyula P, Fischer JP, Kovach SJ (2019). Omental Flap Coverage for Management of Thoracic Aortic Graft Infection. The Annals of thoracic surgery.

